# Discovery and Differential Processing of HLA Class II-Restricted Minor Histocompatibility Antigen LB-PIP4K2A-1S and Its Allelic Variant by Asparagine Endopeptidase

**DOI:** 10.3389/fimmu.2020.00381

**Published:** 2020-03-11

**Authors:** Anita N. Kremer, Judith Bausenwein, Ellie Lurvink, Andreas E. Kremer, Caroline E. Rutten, Cornelis A. M. van Bergen, Sascha Kretschmann, Edith van der Meijden, Maria W. Honders, Daniela Mazzeo, Colin Watts, Andreas Mackensen, J. H. Frederik Falkenburg, Marieke Griffioen

**Affiliations:** ^1^Department of Internal Medicine 5, Hematology/Oncology, Friedrich Alexander University Erlangen-Nuremberg, Erlangen, Germany; ^2^Department of Hematology, Leiden University Medical Center, Leiden, Netherlands; ^3^Department of Internal Medicine 1, Gastroenterology, Pneumology and Endocrinology, Friedrich Alexander University Erlangen-Nuremberg, Erlangen, Germany; ^4^Division of Cell Signaling & Immunology, School of Life Sciences, University of Dundee, Dundee, United Kingdom

**Keywords:** minor histocompatibility antigens, CD4 T-cells, HLA class II, allogeneic stem cell transplantation, graft vs. leukemia effect

## Abstract

Minor histocompatibility antigens are the main targets of donor-derived T-cells after allogeneic stem cell transplantation. Identification of these antigens and understanding their biology are a key requisite for more insight into how graft vs. leukemia effect and graft vs. host disease could be separated. We here identified four new HLA class II-restricted minor histocompatibility antigens using whole genome association scanning. For one of the new antigens, i.e., LB-PIP4K2A-1S, we measured strong T-cell recognition of the donor variant PIP4K2A-1N when pulsed as exogenous peptide, while the endogenously expressed variant in donor EBV-B cells was not recognized. We showed that lack of T-cell recognition was caused by intracellular cleavage by a protease named asparagine endopeptidase (AEP). Furthermore, microarray gene expression analysis showed that PIP4K2A and AEP are both ubiquitously expressed in a wide variety of healthy tissues, but that expression levels of AEP were lower in primary acute myeloid leukemia (AML). In line with that, we confirmed low activity of AEP in AML cells and demonstrated that HLA-DRB1^*^03:01 positive primary AML expressing LB-PIP4K2A-1S or its donor variant PIP4K2A-1N were both recognized by specific T-cells. In conclusion, LB-PIP4K2A-1S not only represents a novel minor histocompatibility antigen but also provides evidence that donor T-cells after allogeneic stem cell transplantation can target the autologous allelic variant as leukemia-associated antigen. Furthermore, it demonstrates that endopeptidases can play a role in cell type-specific intracellular processing and presentation of HLA class II-restricted antigens, which may be explored in future immunotherapy of AML.

## Introduction

HLA-matched allogeneic stem cell transplantation (alloSCT) is a routinely applied treatment option for many hematological malignancies (1). Donor-derived T-lymphocytes can thereby recognize residual malignant cells of the patient, leading to the beneficial graft vs. leukemia (GvL) effect (2). These T-cells are directed against minor histocompatibility antigens, which are polymorphic peptides differentially presented on patient and donor cells that are able to elicit CD8^+^ or CD4^+^ donor T-cells in the context of self-HLA (3). A variety of HLA-class I- and II-restricted minor histocompatibility antigens have been identified by different techniques (4–11), and for several of these antigens, the appearance of specific CD8^+^ T-cells was closely followed by complete remissions of the malignancies (12–15), indicating the clinical relevance of these T-cells.

Different mechanisms have been shown to create HLA class I-restricted minor histocompatibility antigens. Most antigens are encoded by “missense” single-nucleotide polymorphisms (SNPs) in coding gene regions that directly lead to an amino acid change in the protein. Antigens can also be created by SNPs in coding gene regions that are synonymous in the normal reading frame, but missense in an alternative reading frame. In addition, antigens can be derived from proteins that are translated in an alternative reading frame as a result of small indels, such as for LRH-1 (13) or due to the presence of a non-polymorphic alternative start codon as shown for LB-ECGF-1H (14) and LB-ADIR-1F (15). Even SNPs in non-coding gene regions can create polymorphic antigens as a result of alternative mRNA splicing, as reported for PANE1 (16), ACC-6 (17), ITGB2 (18), and TTK (19). Finally, antigens can be encoded by polymorphic genes that are present in the patient, but homozygously deleted in the donor as shown for UGT2B17 (20).

Besides a direct difference in interaction of the presented peptide with the T-cell receptor (TCR), also differential processing of patient and donor variants can determine the immunogenicity of a polymorphism. Intracellular processing has been described to preclude surface presentation of the donor variant of HA-3 (21), HA-8 (22), LB-NUP133-1R (23), and possibly HA-2 (24). These donor variants induce strong T-cell activation after exogenous peptide loading, but fail to activate specific T-cells when endogenously expressed, demonstrating that insufficient processing of the donor variant into the HLA class I pathway is the underlying reason for lack of T-cell recognition. Finally, it has been shown for HA-1 that impaired binding affinity of the donor variant to HLA-A^*^02:01 leads to an increased dissociation and therefore insufficient surface presentation (25).

Here, we present the identification of four new HLA class II-restricted minor histocompatibility antigens by whole genome association scanning (WGAS). For three of these antigens, donor variant peptides were not recognized by specific T-cells when pulsed exogenously on donor EBV-LCL, suggesting that immunogenicity of the patient antigen is attributed to induction of T-cells expressing TCR that are able to discriminate between polymorphic residues. For the remaining antigen, i.e., LB-PIP4K2A-1S, we demonstrate that immunogenicity is at least partially mediated by intracellular cleavage of the donor variant by a protease named asparagine endopeptidase (AEP), thereby precluding its surface presentation. Interestingly, AEP is expressed at low levels in primary acute myeloid leukemia (AML), which hinders differential cleavage of polymorphic peptides and leads to strong T-cell recognition of both patient- and donor-type antigens when presented by HLA class II.

## Materials and Methods

### Hematopoietic Cell Isolation

Peripheral blood was obtained from healthy individuals and patients with leukemia after approval by the Leiden University Medical Center Institutional Review Board and informed consent according to the Declaration of Helsinki. Peripheral blood mononuclear cells (PBMC) were isolated by Ficoll-Isopaque separation and cryopreserved. CD34+ hematopoietic stem cells were isolated from stem cell grafts and CD33+ AML blasts from peripheral blood or bone marrow by flow cytometric cell sorting.

### Cell Culture

MJS, SD1 (26), and EBV-transformed B-cell lines (EBV-LCL) were cultured in IMDM (Lonza BioWhittaker) supplemented with 10% fetal calf serum (FCS) (Cambrex), 1% penicillin/streptomycin (Lonza), and 1.5% L-glutamine (Lonza). T-cell clones were cultured in IMDM supplemented with 5% human AB serum (Anprotec), 5% FCS, and 100 IU/ml interleukin-2 (IL-2) (Chiron), and restimulated every 10–20 days with irradiated (50 gray) allogeneic PBMCs and 0.8 μg/ml phytohemagglutinin (PHA) (Oxoid).

### Whole Genome Association Scanning

WGAS was performed as previously described (27). Briefly, a panel of 80 EBV-LCL was genotyped for 1.1 million SNPs using microarrays. T-cell recognition of the panel was measured after retroviral transduction with the HLA-DRB1^*^03:01 restriction molecule, and recognition patterns were compared with SNP genotype data. The level of matching between the patterns of T-cell recognition and SNP genotypes was calculated by Fisher's exact test using PLINK WGA analysis software (28). For WGAS, test results needed to be categorized in two distinct groups. Therefore, EBV-LCL were divided into positive and negative groups based on the level of IFN-γ production. WGAS was performed by combining T-cell recognition with SNP genotyping data.

### Sequencing of PIP4K2A Genotype

Genomic DNA of patient- and donor-derived EBV-LCL, MJS, primary AML, and primary healthy CD34^+^ hematopoietic stem cells was isolated by using QIAamp DNA Blood mini kit (Qiagen) mini columns. DNA concentrations have been measured using a NanoDrop Microvolume spectrophotometer (Thermo Fisher). PCR amplification (initial denaturation 2 min 30 s; 94°C; 40 cycles: denaturation 45 s; 95°C, annealing 45 s; 60°C, elongation 1 min 30 s; 72°C; final elongation 10 min, 72°C) was performed using 100 ng of genomic DNA with the following primers: 5′-GCC AAA GAA CTG CCA ACT CT-3′ (forward primer) and 5′-GGC CTC TCC ACT GAC TGT TC-3′ (reverse primer). PCR products were purified using the QIAquick PCR purification kit (Qiagen). Sanger sequencing was performed using 25 pmol of the forward and reverse primer, respectively, and 200 ng of purified PCR product. Sequence reaction was performed by Eurofins Genomics (Luxembourg).

### Flow Cytometry

For isolation of retrovirally transduced cells carrying the marker gene CD2, APC-labeled anti-murine CD2 (clone RM2-5; Biolegend) was used. Healthy hematopoietic stem cells were isolated based on expression of CD34 using PE-labeled anti-CD34 (8G12; BD) and acute myeloid leukemic cells were isolated based on expression of CD33 using BV421-labeled anti-CD33 (WM53; BD). Cells were washed twice with phosphate-buffered saline containing 2% FCS and incubated with fluorochrome-conjugated monoclonal antibodies for 30 min at room temperature. Data acquisition was performed on a fluorescence-activated cell sorter Canto II and a fluorescence-activated cell sorter BD FACS Aria (BD Biosciences). Forward scatter/side scatter was used for gating on viable cells. FSC-H/-A was used for doublet exclusion. Data were analyzed with Kaluza 2.1. (Beckman Coulter).

### Retroviral Constructs and Transduction

All experiments involving retroviral vectors were approved by the government and handled according to biosafety level 2. All constructs were cloned in MP71 retroviral vectors containing different marker genes. PIP4K2A was linked to the GFP marker gene, AEP to murine ΔCD2, and HLA-DRB1^*^03:01 to ΔNGFR. All constructs were verified by sequencing. Retroviral supernatant was obtained by transfecting wild-type Φnx-A packaging cells as previously described (29), with the exception that the X-tremeGENE HP DNA Transfection Reagent transfection kit (Roche Diagnostics) was used. Non-tissue culture-treated culture plates were coated (overnight at 4°C) with RetroNectin (30 μg/ml) Recombinant Human Fibronectin Fragment (Takara) before harvested retroviral supernatants were applied and centrifuged at 32°C for 2 h at 2,000 *g*. After centrifugation, cells (1–5 × 10^5^) were directly transferred into the infectious supernatant and marker gene expression measured after 3 days by flow cytometry.

### Antigen Presentation Assays

Stimulator cells (3 × 10^4^ cells/well or as indicated) were co-incubated with the CD4^+^ T-cell clone (5 × 10^3^ cells/well or as indicated) overnight at 37°C in 96-well plates in duplicates. For peptide loading, stimulator cells were incubated with indicated peptide concentrations for 2 h at 37°C before T-cell clones were added. For shutdown of the tet-off system, cells were cultured in the presence of 50 μg/ml doxycycline and washed twice before co-incubation with the T-cell clone. Cytokine release was measured after overnight incubation in 100-μl supernatants by IFN-γ ELISA following the instructions of the manufacturer (Thermo Fisher Scientific).

### Enzymatic Activity of AEP

To determine the activity of AEP, cells were lysed by three cycles of freezing and thawing. Subnuclear fractions were collected by harvesting supernatants after centrifugation at 10,000 *g* for 20 min. Protein concentrations were measured using the BCA protein assay (Thermo Scientific). Cellular lysates (2 and 5 μg) were resuspended in sodium citrate buffer (50 mM, pH 5.5; 5 mM DTT, 0.1% CHAPS). Z-Ala-Ala-Asn-AMC (10 μM; Bachem) was added to the lysates for 30 min at room temperature. Developing fluorescence (excitation 370 nm; emission 460 nm) was measured for 10 min on a NOVOstar analyzer (BMG labtech).

### Microarray Gene Analysis

Total RNA was isolated using small- and micro-scale RNAqueous isolation kits (Ambion) and amplified using the TotalPrep RNA amplification kit (Ambion). After preparation using the whole-genome gene expression direct hybridization assay (Illumina), cRNA samples were dispensed onto Human HT-12 v3 Expression BeadChips (Illumina). Hybridization was performed in the Illumina hybridization oven for 17 h at 58°C. Microarray gene expression data were analyzed using R 2.15. Normalization was done in the lumi package, using the variance stabilizing transformation and quantile normalization (30).

### Statistical Analysis

Data were analyzed with Prism 8.3.0 (GraphPad Software Inc.). If not otherwise stated, for statistical analysis, at least three individual experiments were performed and the unpaired *t*-test was applied. Statistical significance was indicated as ^*^P < 0.05 or ^**^P < 0.01. For WGAS, the level of matching between T-cell recognition pattern and SNP data was calculated according to Fisher's exact test.

## Results

### Identification of Four New HLA Class II-Restricted Minor Histocompatibility Antigens by WGAS

**Figure 1:**
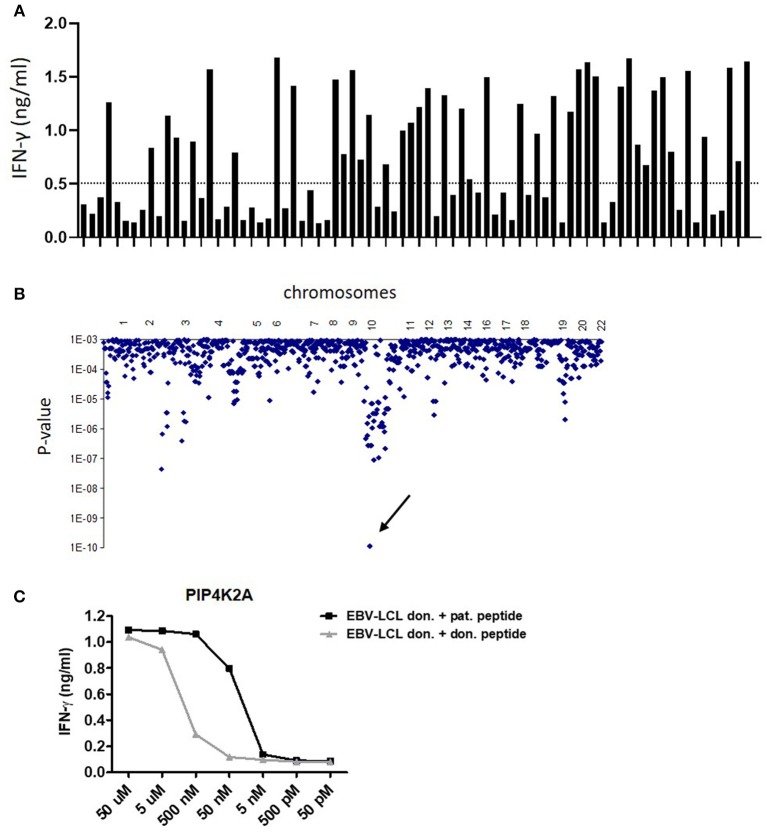
Identification of LB-PIP4K2A-1S as new HLA class II-restricted minor histocompatibility antigen by whole genome association scanning. **(A)** T-cell recognition of a panel of 80 HLA-DRB1^*^0301 transduced EBV-LCL. Bars represent the level of IFN-γ (ng/ml) in ELISA released by clone 100 upon co-incubation with the different EBV-LCL. **(B)** Whole genome association scanning of the recognition data for 80 HLA-DRB1^*^0301 transduced EBV-LCL and the corresponding SNP data revealed one strongly correlating missense SNP in PIP4K2A (rs10828317) (arrow). The *y*-axis indicates *P-*values for the significance of association between SNPs and T-cell recognition of EBV-LCL. The *x*-axis represents the 1 million tested SNPs as distributed over the human chromosomes. **(C)** Synthetic peptides of patient and donor variants were loaded on donor EBV-LCL in indicated concentrations and recognition by T-cell clone 100 was measured in IFN-γ ELISA. The LB-PIP4K2A-1S-specific T-cell clone 100 also showed recognition of the donor PIP4K2A-1N variant at peptide concentrations over 50 nM.

The target antigens of four CD4^+^ T-cell clones were identified by WGAS. All T-cell clones have been shown to be specific for minor histocompatibility antigens by recognizing patient but not donor EBV-LCL. Clone 100 has been isolated from bone marrow of patient 3,087, 5 weeks after donor lymphocyte infusion (DLI) for relapsed chronic myeloid leukemia (CML) after alloSCT (9) and was restricted to HLA-DRB1^*^03:01. Clone 8-10A and clone 8-15 were isolated from peripheral blood of patient 2,877, 4 weeks after DLI for relapsed CML after alloSCT and were both restricted to HLA-DQB1^*^06:02. Finally, clone 15-18, which was also HLA-DQB1^*^06:02-restricted, was isolated from patient 5,852 who was treated with DLI for mixed chimerism 6 months after alloSCT for myelodysplastic syndrome refractory anemia with excess of blasts type 2. To identify the target antigens of these T-cell clones, we tested reactivity against a panel SNP-genotyped EBV-LCL either transduced with HLA-DRB1^*^03:01 (clone 100; Figure 1A) or endogenously expressing HLA-DQB1^*^06:02 (clones 8-10A, 8-15, and 15-18) and correlated T-cell recognition data with SNP genotypes of the respective EBV-LCL (27). The level of matching was calculated according to Fisher's exact test.

For clone 100, one SNP genotype significantly associated with T-cell recognition with a *P*-value <1 × 10^−9^ (Figure 1B). This was a coding missense SNP (rs10828317) in PIP4K2A (HGNC ID: 8997). Sequencing cDNA of patient and donor EBV-LCL for the region encompassing the SNP confirmed heterozygosity in patient cells at this position (G/A), whereas donor cells were homozygous A/A. This A-to-G transition creates an Asn-to-Ser substitution at amino acid position 251 (N->S251) of the PIP4K2A protein.

For clone 8-10A, two SNPs (rs4242391 and rs1133782) significantly associated with T-cell recognition with *P*-values <1 × 10^−9^. One of these SNPs (rs1133782) is a missense variant in TNFRSF10D (HGNC ID: 11907). The T-to-C transition creates a Leu-to-Ser substitution at amino acid position 310 (L->S310) of the TNFRSF10D protein.

Two SNP genotypes (rs4740 and rs4905) with *P*-values <1 × 10^−8^ associated with T-cell recognition by clone 8-15. Rs4740 is a missense variant in EBI3 (HGNC ID: 3129) and the G-to-A transition translates into a Val-to-Ile substitution at amino acid position 201 (V->I201) of the EBI3 protein.

Also for clone 15-18, two SNP (rs17700475 and rs6441226) associated with T-cell recognition with *P-*values <1 × 10^−9^. SNP rs6441226 is an intron variant in MFSD1 (HGNC ID: 25874), which has a minor allele frequency (MAF) of 0.1478 in 1,000 Genomes. Searching the SNP database for a missense SNP with a similar MAF revealed SNP rs28364680 that encodes a C-to-T transition leading to a Pro-to-Ser substitution.

T-cell recognition of donor EBV-LCL loaded with synthetic peptides surrounding the patient and donor variants confirmed T-cell specificity for the patient variants and validated these peptides as minor histocompatibility antigens (Supplemental Table 1). T-cell clones 8-10A, 8-15, and 15-18 failed to recognize donor peptide variants (Supplemental Figure 1). T-cell clone 100, however, showed strong recognition of the donor variant (INEGQKIYIDDN**N**KKVFLE) at peptide concentrations >50 nM, while there was no recognition at lower peptide concentrations (Figure 1C). The patient peptide variant (INEGQKIYIDDN**S**KKVFLE) was recognized at peptide concentrations >5 nM. These data indicate that the TCR of clone 100 is less able to discriminate between polymorphic peptides than the TCR of clones 8-10A, 8-15, and 15-18, suggesting that also other mechanisms are involved in differential recognition of patient and donor EBV-LCL.

### Surface Presentation of Donor Variant PIP4K2A-1N Is Hampered by AEP-Mediated Cleavage

**Figure 2:**
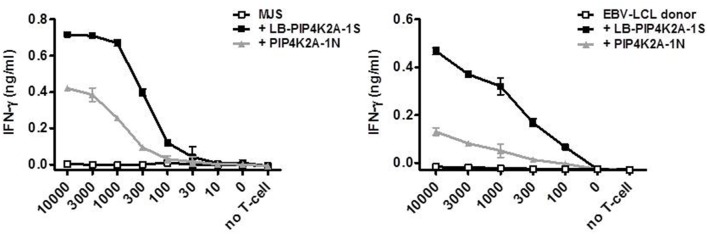
Cell type-specific recognition of endogenously expressed PIP4K2A-1N donor variant. Full-length genes encoding LB-PIP4K2A-1S and PIP4K2A-1N were retrovirally transduced in MJS **(left)** or donor EBV-LCL **(right)** and transduced cells were isolated by flow cytometric sorting based on expression of marker gene GFP. T-cell recognition by clone 100 of the different cell lines was measured in IFN-γ ELISA. Indicated is the release of IFN-γ (ng/ml) by T-cell clone 100 upon co-incubation with indicated numbers of transduced MJS cells or EBV-LCL. Indicated are mean and standard deviation of duplicate wells. One representative of three independent experiments is shown.

**Figure 3:**
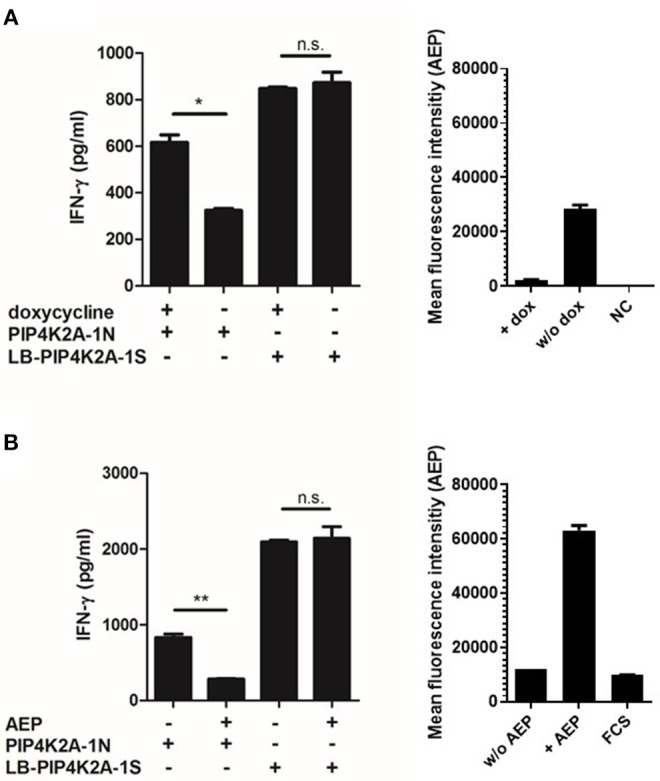
Recognition of endogenously expressed PIP4K2A-1N donor variant correlates with activity of asparagine endopeptidase AEP. **(A)** SD1 cells expressing a tet-off vector system with AEP were retrovirally transduced with full-length genes encoding the LB-PIP4K2A-1S patient variant or PIP4K2A-1N donor variant and tested for T-cell recognition with and without prior doxycyclin (dox) treatment (left). Enzyme activity with and without doxycyclin treatment has been measured in cellular lysates (right). NC, negative control. **(B)** T-cell recognition of MJS retrovirally transduced with full-length genes encoding LB-PIP4K2A-1S patient variant or PIP4K2A-1N donor variant with or without additional transduction of AEP (LGMN). T-cell recognition has been tested in IFN-γ ELISA (left). Enzyme activity of AEP has been measured in transduced cell lines and fetal calf serum (FCS) as negative control (right). ^*^P < 0.05; ^**^P < 0.01 (unpaired *t*-test). One representative of three independent experiments is shown.

To investigate whether donor EBV-LCL fail to present the donor variant peptide as a consequence of low gene expression, we established overexpression of PIP4K2A by retroviral transduction. Interestingly, overexpression of full-length PIP4K2A encoding donor variant PIP4K2A-1N induced only low recognition of donor EBV-LCL, while significant recognition was observed for transduced melanoma Mel-Juso (MJS) cells (Figure 2). This suggests that another intracellular mechanism than gene expression is involved in differential recognition of LB-PIP4K2A-1S and its allelic variant. As the donor PIP4K2A-1N variant is defined by an asparagine in substitution of a serine in the patient variant, we hypothesized that the donor peptide may be intracellularly cleaved by AEP (alias LGMN) as described for other antigens (26). We therefore expressed the full-length patient and donor genes for PIP4K2A in melanoma cell line SD1, which expressed a tet-off system with AEP under control of doxycycline (26). Testing T-cell recognition of these transduced SD1 cell lines revealed increased recognition of PIP4K2A-1N after treatment with doxycycline, which shuts down AEP enzyme activity (Figure 3A). To confirm these data, we cloned the AEP gene and retrovirally overexpressed the protein in MJS cells co-transduced with the PIP4K2A variants. T-cell recognition of PIP4K2A-1N was significantly decreased upon overexpression of AEP, while recognition of LB-PIP4K2A-1S was not affected (Figure 3B). These data indicated that donor variant PIP4K2A-1N is enzymatically cleaved by AEP, thereby hampering presentation and T-cell recognition of the epitope at the cell surface.

### Low Expression of AEP in Primary AML and Healthy CD34^+^ Hematopoietic Stem Cells

**Figure 4:**
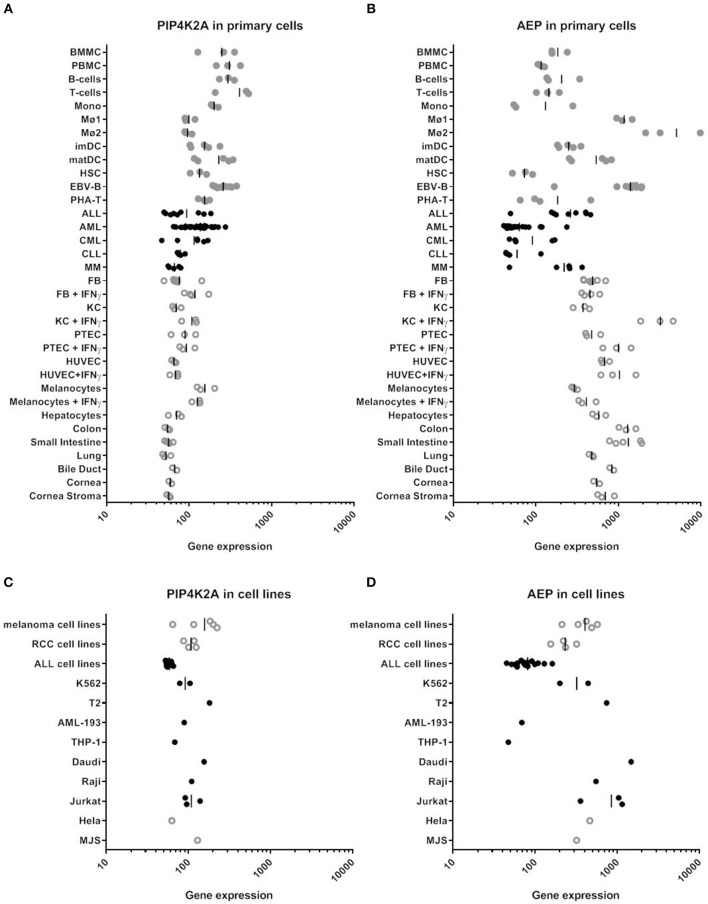
RNA expression of PIP4K2A and AEP in various human cell types by microarray gene analysis. **(A)** PIP4K2A gene expression in primary cells. **(B)** AEP gene expression in primary cells. **(C)** PIP4K2A gene expression in cell lines. **(D)** AEP gene expression in cell lines. Healthy and malignant hematopoietic cells are represented by gray and black dots, respectively, while open symbols represent non-hematopoietic cell types. Gene expression was measured on Illumina HT-12 BeadChips as described previously (30). BMMC, bone marrow mononuclear cells; PBMC, peripheral blood mononuclear cells; Mono, monocytes, Mø1, type 1 macrophages; Mø2, type 2 macrophages; imDC, immature dendritic cells; matDC, mature dendritic cells; HSC, hematopoietic CD34^+^ stem cells; EBV-B, EBV-transformed B cells; PHA-T, PHA-stimulated T-cells; ALL, acute lymphocytic leukemia; AML, acute myeloid leukemia; CML, chronic myeloid leukemia; MM, multiple myeloma; FB, fibroblasts; KC, keratinocytes; PTEC, proximal tubular epithelial cells; HUVEC, human umbilical vein endothelial cells.

We hypothesized that enzymatic cleavage of donor variant PIP4K2A-1N could potentially be overcome by high expression of the substrate, i.e., PIP4K2A, or low expression or activity of AEP, and therefore analyzed mRNA expression of PIP4K2A and AEP by microarray gene analysis (Figure 4 and Supplemental Table 2). In addition to MJS and various other cancer cell lines, we analyzed gene expression in a wide variety of primary cell types of hematopoietic and non-hematopoietic origin, including bone marrow and PBMC, primary T- and B-cells, monocytes, macrophages, monocyte-derived immature and mature dendritic cells, healthy CD34^+^ hematopoietic stem cells, acute and chronic myeloid leukemia, acute and chronic lymphocytic leukemia, and multiple myeloma as well as primary fibroblasts, keratinocytes, proximal tubular epithelial cells, human umbilical vein endothelial cells, melanocytes, hepatocytes, and gut, lung, bile duct, and cornea epithelial cells. Various non-hematopoietic cell types were also cultured in the absence or presence of IFN-γ to mimic an inflammatory environment. Interestingly, we observed low expression of AEP especially in primary AML and CD34^+^ hematopoietic stem cells, while PIP4K2A expression was less variable.

### Primary AML Elicit T-Cell Reactivity Independent of Their SNP Status

**Figure 5:**
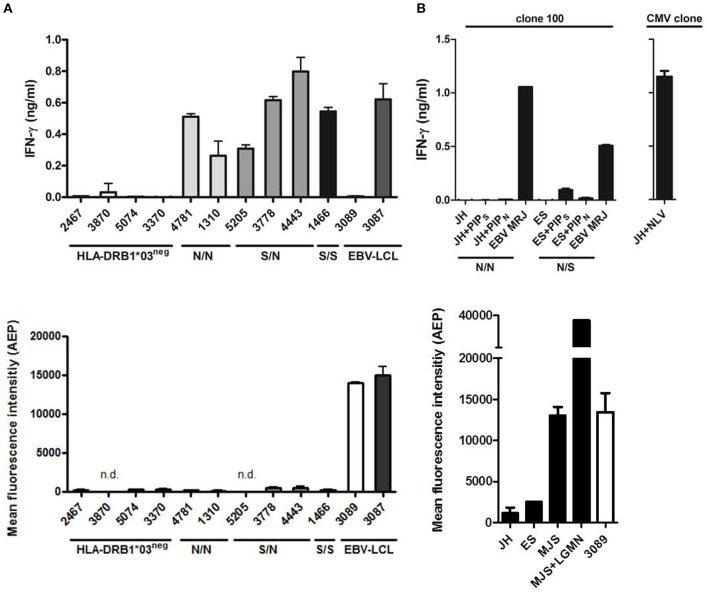
Primary human AML are recognized independent of the SNP for PIP4K2A. **(A)** Recognition of primary human AML by T-cell clone 100 as tested in IFN-γ ELISA (top panel). PIP4K2A genotypes [N/N (light gray), S/N (dark gray), or S/S (black)] are depicted for HLA-DRB1*03 positive primary AML. EBV-LCL 3087 is derived from the patient from whom T-cell clone 100 has been isolated, and EBV-LCL 3089 is from its stem cell donor. Enzyme activity of AEP has been measured in corresponding AML cell lysates (bottom). n.d., not determined. **(B)** T-cell recognition of primary human CD34^+^ cells has been tested by IFN-γ ELISA (top panel). PIP4K2A genotypes (N/N, S/N, or S/S) are depicted. Both patients are typed positive HLA-DRB1*03:01, patient JH is in addition typed positive for HLA-A*02:01 and viability of CD34^+^ cells is confirmed by recognition of CMV peptide NLV in HLA-A*02:01. Enzyme activity of AEP in CD34 cell lysates is depicted (bottom). One representative of two independent experiments is shown.

Since AEP expression is low in primary AML and CD34^+^ hematopoietic progenitor cells, we tested whether T-cell clone 100 could recognize PIP4K2A peptides on these cell types independent of the patient SNP. Primary AML cells were sorted on CD33 by flow cytometry and tested for recognition by T-cell clone 100 in IFN-γ ELISA. Data showed that all AML that were positive for the relevant restriction molecule HLA-DRB1^*^0301 were recognized by the T-cell clone independent of their SNP status (Figure 5A). In contrast, AML cells that lacked HLA-DRB1^*^0301 were not recognized. We also tested activity of AEP in these cells and confirmed low or absent enzyme activity, which is in line with low mRNA expression in these cells. In addition to AML cells, T-cell recognition of primary CD34^+^ hematopoietic progenitors was tested. In contrast to AML, donor variant PIP4K2A-1N was not recognized on CD34^+^ cells, which is probably due to low HLA class II expression or other accessory molecules in these cells since exogenous peptide loading also induced only marginal T-cell recognition of LB-PIP4K2A-1S. In contrast, a CMV derived antigen peptide presented in HLA-A^*^02:01 mediated strong T-cell recognition (Figure 5B). In conclusion, the data showed that T-cell clone 100 recognizes primary AML independent of their SNP status, thereby confirming surface presentation of donor variant PIP4K2A-1N in the absence of AEP.

## Discussion

T-cells directed against minor histocompatibility antigens are well established to mediate strong immune responses both against residual malignant cells and potentially against healthy non-malignant cells of the patient. A further understanding of these T-cell responses is crucial for eventually separating the effects of GvL and Graft vs. host disease. We here present four new HLA class II-restricted minor histocompatibility antigens identified by WGAS. Furthermore, we demonstrate that one of the new antigens is differentially presented on patient and donor cells due to intracellular cleavage of the donor peptide by AEP. This enzyme is not or less active in AML due to low expression of the endopeptidase. As a result, AML cells can be targeted by donor T-cells independent of the SNP status. These data illustrate that endopeptidases can play a role in cell type-specific intracellular processing and presentation of HLA class II antigens and provide evidence that after alloSCT, donor T-cells for minor histocompatibility antigens can also target the respective allelic variants as cell type-specific autoantigens.

Although it has been shown for various HLA class I-restricted minor histocompatibility antigens that their immunogenicity is based on differential intracellular processing of patient and donor variant peptides (21, 22, 24), all so far identified HLA class II-restricted minor histocompatibility antigens are recognized by T-cell receptors that fail to react with donor variants. Also, for three new HLA class II antigens identified here, donor variants are not recognized when loaded as exogenous peptides, indicating that patient and donor peptides are differentially recognized by the T-cell receptor as expressed by the specific T-cell clone. Alternatively, it is also possible that the donor peptide does not sufficiently bind to the HLA molecule and is therefore not presented, as described for HA-1 (25). However, for one new HLA class II antigen, i.e., LB-PIP4K2B-1S, we observed that its donor variant PIP4K2A-1N is strongly recognized when pulsed as exogenous peptide, suggesting involvement of an intracellular processing mechanism in differential recognition of patient and donor variant. Of note, peptide titration still showed a marked difference in T-cell activation between LB-PIP4K2A-1S and its donor variant, indicating that intracellular processing is not the only mechanism and that the affinity of the T-cell receptor as expressed by the specific T-cell clone also contributes to differential recognition of patient and donor peptides. However, the observation that AEP strongly affects endogenous presentation of donor variant PIP4K2A-1N, but not of LB-PIP4K2A-1S, confirmed involvement of this endopeptidase in intracellular processing of the HLA class II antigen.

A role for AEP in antigen processing has already been described for other antigens. For tetanus toxin C fragment (TTCF), it has been demonstrated that deamidation of an asparagine residue hinders enzymatic cleavage by AEP, thereby perturbing antigen presentation (31). Whereas cleavage by AEP enhances antigen presentation for TTCF, presentation of LB-PIP4K2A-1S is disrupted. Similarly, it has been shown for myelin basic protein (MBP), which is an autoantigen in the inflammatory demyelinating disease multiple sclerosis, that autoreactive T-cells can evade central tolerance due to enzymatic cleavage of the autoantigen in the thymus (26). It has been suggested that under certain circumstances, these MBP-specific T-cells may become activated and induce autoimmunity. One of these circumstances may be the posttranslational modification of the asparagine residue by deamidation, thereby hindering cleavage and enhancing MBP antigen presentation. Non-enzymatic deamidation of asparagine to aspartic acid is the most commonly observed posttranslational modification in proteins. Although the C-terminally flanking lysine in PIP4K2A-1N is not ideal, deamidation of the asparagine can occur at intermediate turnover rates (32) and may therefore contribute to surface presentation of the donor variant.

Apparently, in the stem cell donor for patient 3,087, a T-cell expressing a TCR for donor variant PIP4K2A-1N has evaded central tolerance, suggesting that the donor variant is not expressed during thymic selection. This is in line with our observation that even non-physiological levels of overexpression of the donor variant does not lead to strong T-cell activation. This, together with the observation that the stem cell donor and the transplanted patient lack any signs of autoimmunity, indicates that donor variant PIP4K2A-1N is not or not sufficiently presented on all or the majority of healthy tissues *in vivo*.

Thinking about therapeutic approaches, it is tempting to speculate that the TCR as expressed by the T-cell clone for LB-PIP4K2A-1S could be used for gene therapy to treat AML in patients who are homozygous for the donor variant (N/N) without need of prior transplantation. In this setting, the TCR is expected to selectively recognize PIP4K2A-1N as tumor antigen on low AEP-expressing leukemic cells, whereas all or the majority of healthy tissues are not expected to present the epitope due to intracellular AEP-mediated cleavage. While low expression of AEP in healthy CD34^+^ hematopoietic stem cells is of concern, our *in vitro* experiments failed to show any T-cell recognition of these cells, probably due to a low overall HLA class II expression or lack of other accessory molecules stimulatory capacity in these cells. Moreover, we isolated the T-cell clone for LB-PIP4K2A-1S during GvL reactivity from a patient who was transplanted with CD34^+^ hematopoietic stem cells from a PIP4K2A-1N homozygous donor, but had no signs of myeloablation. However, it cannot entirely be excluded that side effects may occur due to presentation of PIP4K2A-1N on certain cell types or healthy tissues as a result of low AEP expression or posttranslational modification of the asparagine residue. Although it is tempting to speculate, we would like to emphasize that we do not present PIP4K2A-1N as ideal target for immunotherapy of AML, since that conclusion requires additional *in vitro* and *in vivo* experiments, but rather as proof of concept that endopeptidases play a similar role in cell type-specific intracellular processing of HLA class II-restricted minor histocompatibility antigens as intracellular processing is known to be involved in presentation of HLA class I-restricted minor histocompatibility antigens (21–23).

Another question is why AML cells express low amounts of AEP and whether this can be influenced. Intriguingly, it was recently described that AEP is downregulated by PD-1 signaling in regulatory T-cells (33). It is tempting to speculate that this may also occur in tumor cells during immune evasion (34). This would make PIP4K2A-1N more attractive as a leukemia-associated antigen to be targeted by immunotherapy perhaps after or during treatment with cisplatin, a chemotherapeutic agent that is known to induce deamidation (35). Altogether, convincing evidence that PIP4K2A-1N can be used as a leukemia-specific target is lacking and demands further exploration using *in vivo* models. Nevertheless, discovery of LB-PIP4K2A-1S as a new minor histocompatibility antigen revealed an interesting role of an endopeptidase in cell type-specific intracellular processing of this HLA class II-restricted antigen and its donor variant, which may be explored in future immunotherapy of AML.

## Data Availability Statement

The analyzed datasets for this study are deposited in the NCBI's Gene Expression Omnibus and are accessible through GEO Series accession number GSE76340 (http://www.ncbi.nlm.nih.gov/geo/query/acc.cgi?acc=GSE76340).

## Ethics Statement

The studies involving human participants were reviewed and approved by Leiden University Medical Center Institutional Review Board. The patients/participants provided their written informed consent to participate in this study.

## Author Contributions

ANK performed and designed research and wrote the manuscript. JB, EL, CR, CB, SK, DM, EM, and MH performed research. AEK, CW, AM, JF, and MG designed research and wrote/reviewed the manuscript.

### Conflict of Interest

The authors declare that the research was conducted in the absence of any commercial or financial relationships that could be construed as a potential conflict of interest.
