# Supplementary material for: Discovery and Differential Processing of HLA Class II-Restricted Minor Histocompatibility Antigen LB-PIP4K2A-1S and Its Allelic Variant by Asparagine Endopeptidase
Source: Front Immunol. 2020 Mar 11;11:381. doi: 10.3389/fimmu.2020.00381 (PMC7078166; doi:10.3389/fimmu.2020.00381)
Supplement: Supplementary file 1 [file Data_Sheet_1.PDF]

Supplemental Figure 1:

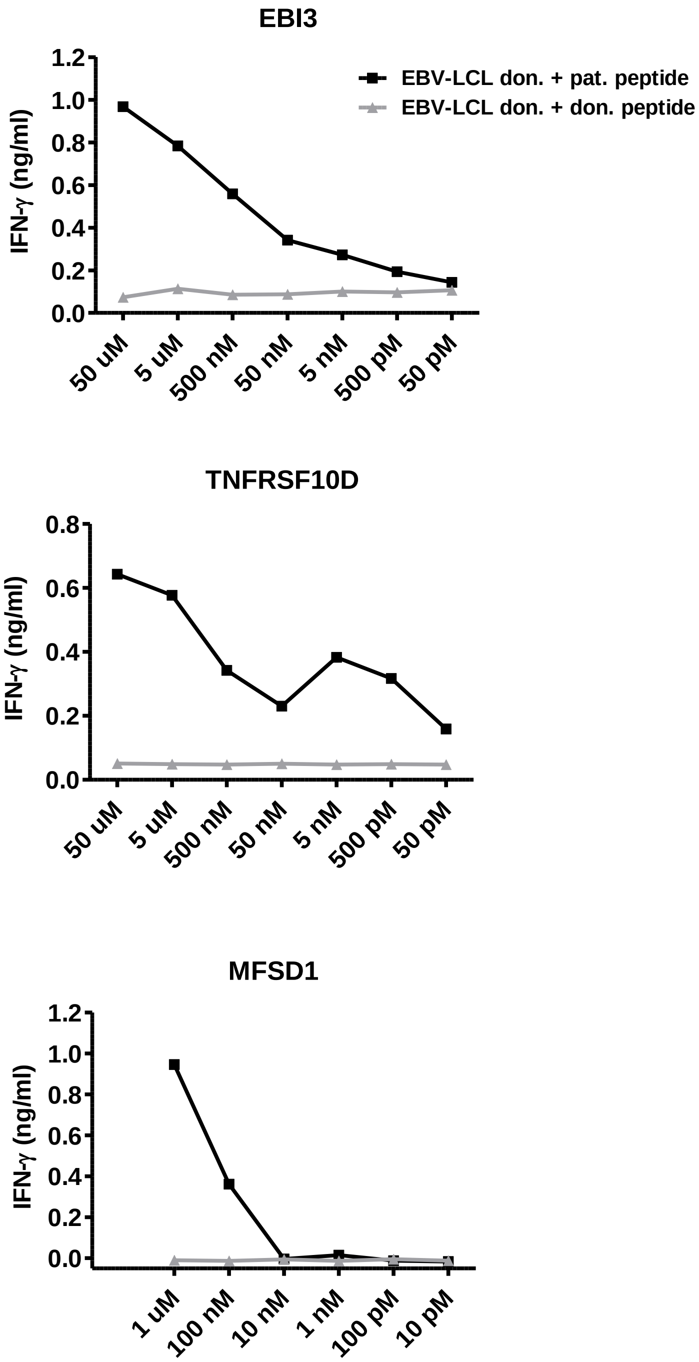

Identification of three new HLA class II restricted minor histocompatibility antigen by WGAS: Synthetic peptides of patient and donor variants for three newly identified minor antigens were loaded on donor EBV-LCL in indicated concentrations and recognition by the respective T-cell clones was measured in IFN-γ ELISA. T-cell clones for LB-TNFRSF10D-1S, LB-EBI3-1I and LB-MFSD1-1S strongly recognized the patient variant peptide, but failed to recognize the respective donor variants even at high peptide concentrations.

Supplemental Table 1. Characteristics of four newly identified minor antigens.

clone	patient	HLA restriction	MiHA	Patient epitope	Donor variant	SNP ID
100	3087	DRB1*0301	LB-PIP4K2A-1S	INEGQKIYIDDN**S**KKVFLE	INEGQKIYIDDN**N**KKVFLE	rs10828317
8-10A	2877	DQB1*0602	LB-TNFRSF10D-1S	AELTGVTVE**S**PEEPQ	AELTGVTVE**L**PEEPQ	rs1133782
8-15	2877	DQB1*0602	LB-EBI3-1I	RPRARYY**I**QVA	RPRARYY**V**QVA	rs4740
15-18	5852	DQB1*0602	LB-MFSD1-1S	RGA**S**AAPGALPALC	RGA**P**AAPGALPALC	rs28364680

Supplemental Table 2. *PIP4K2A* and *AEP* gene expression in cells of different origins[1].

Hematopoietic cells			Hematol malignancies			Non-hematopoietic cells			Malignant cell lines		
	PIP4K2	AEP		PIP4K2A	AEP		PIP4K2A	AEP		PIP4K2A	AEP
BMMC	263	157	ALL	130	166	FB	71	460	melanoma	201	575
	128	241		80	462		69	703		116	489
	356	158		52	404		143	378		224	213
PBMC	215	115		50	408		50	558		64	420
	293	106		72	249		66	488		184	334
	419	128		184	50		72	382	RCC	118	321
B-cells	233	142		65	177		65	453		87	222
	299	136		60	306	FB+IFNγ	107	389		124	235
	354	340		152	155		103	360		101	155
T-cells	208	139	AML	80	60		88	596	ALL	65	81
	527	102		157	45		173	468		62	71
	494	193		207	238	KC	66	392		56	52
Mono	192	58		90	55		63	283		59	91
	186	54		65	44		80	448		53	82
	226	282		69	49	KC+IFNγ	120	1869		60	109
mø type I	118	954		162	69		123	3210		60	61
	91	1114		103	46		81	4640		54	59
	89	1475		91	48	PTEC	88	402		56	73
mø type II	108	2144		90	45		61	415		55	130
	91	9879		138	75		120	611		58	99
	89	3218		82	46	PTEC+IFNγ	77	948		66	57
imDC	173	285		123	55		84	650		62	61
	153	184		130	82		118	1430		57	68
	237	191		107	53	HUVEC	69	784		62	76
	105	353		155	53		66	621		60	45
	103	245		102	45		62	642		53	162
matDC	341	639		187	41	HUVEC+IFNγ	73	1640	K562	78	200
	261	271		159	51		73	851		104	443
	303	827		138	114		58	617	T2	181	745
	128	711		188	48	Melanocytes	204	273	AML-193	89	69
	116	255		149	43		125	321	THP-1	68	47
HSC	135	92		197	50		138	291	Daudi	155	1480
	103	75		277	47	Melan+IFNγ	135	332	Raji	110	554
	163	52		102	61		108	539	Jurkat	95	1152
EBV-B	262	963		154	119		135	364		91	1047
	217	1669		118	47	Hepatocytes	81	488		139	358
	286	1362		227	46		56	705	Hela	63	465
	377	1449	CML	47	57		74	547	MJS	129	320
	329	1486		169	57	Colon	56	1019			
	315	1245		126	159		58	1243			
	221	1635		127	48		51	1632			
	194	168		72	56	Small Intestine	54	1855			
	199	1921		152	170		64	1141			
	212	1900	CLL	90	115		58	937			
	246	1645		72	44		57	783			
PHA-T	177	113		80	48		51	1930			
	129	97		75	46	Lung	60	488			
	159	465		75	44		50	442			
	151	65	MM	56	48		48	501			
				57	254	Bile Duct	71	791			
				79	364		63	882			
				76	179	Cornea	54	899			
				66	259		59	562			
							57	629			
						Cornea Stroma	57	594			
							62	503			

[1]Gene expression for *PIPK42A* and *AEP* as measured on Illumina HT-12.0 microarrays by probes ILMN_2152465 and ILMN_1698019, respectively. Data were obtained from NCBI's Gene Expression Omnibus through GEO Series accession number GSE76340 (http://www.ncbi.nlm.nih.gov/geo/query/acc.cgi?acc=GSE76340).
